# Vale

**Published:** 1891-05

**Authors:** 


F. E. DANIEL, M. D., EDITOR AND PUBLISHER.

THIS JOURNAL, by unanimous vote of the members, was made the Official Organ of the Austin District Medical Society.

COLLABORATORS:

*Dr. R. M. Swearingen, Austin.*
*Prof. B. E. Hadra, M. D., Galveston.*
*Prof. Geo. Cupples, M. D., San Antonio.*
*Dr. T. C. Osborn, Cleburne, Texas.*
*Dr E. J. Doering, Chicago.*
*Dr. E. J. Beall, Fort Worth.*
*Dr Odo Betz, Germany.*
*Prof. W. B. Rogers, M. D., Memphis.*
*Dr. J. W. McLaughlin, Austin.*
*Dr. T. J. Tyner, Austin.*
*Prof. J. F. Y. Paine, M. D., Galveston.*
*Dr. R. H. L. Bibb, Mexico.*
*Dr. T. J. Bennett. Austin.*
*Dr. Bat Smith, Wharton.*
*Dr. E. Meierhof, New York.*
*L. H. Luce, M. D., West Tisbury, Mass.*

### VALE.

In resigning, a second time, from the office of Secretary of the Texas State Medical Association, a position to which the appreciation and kind partiality of the *majority* had twice elected us, we wish to make our grateful acknowledgments to the large number of true-hearted physicians who have, "through evil as well as through good report," honored us by their confidence and support; and to say that in doing so we were actuated by the purest and most unselfish motives,—the preservation of harmony in an organization which we have, for years, labored to build up and advance. In so doing, we made a financial sacrifice which we can ill afford; but principle on our part, and peace in the guild, were, with us, higher considerations than self.

Not a word of complaint, so far as we are aware, has ever been made as to the business portion of our long service; but, as editor of an independent journal, devoted, heart and soul to the good of legitimate medicine and the defense of the dignity of professional character, we had set up a high standard of excellence, and insisted upon an exact conformity to it; insisted upon a literal compliance with every requirement of the code, and that all who were admitted to the brotherhood should toe the mark, so to speak, of purity of professional life; and whenever any have departed therefrom, to our knowledge, or made any infraction of the code, as we conceived, the JOURNAL has rapped their toes without hesitation, and in no uncertain manner. The right to do this was not, by some, conceded to *a paid officer* of the Association; and as we conceived it to be a bounden duty, though self-imposed, it will readily be seen that self-respect, if no other consideration, would not permit us to consent to be thus hampered,—hence our positive refusal to serve longer as Secretary.

We were not willing to occupy a position where even the most prejudiced, or any who might feel that their toes had been rapped, could say that we were an element of discord, or an impediment to the wheels of progress.

In denouncing the sale of Hepatic Pills, under a printed label, bearing the name of an ex-President, we were sincere in believing that we were doing right in defending the dignity of the Association from an outrage. The Association did not see proper to so regard it, and exonerated the proprietor from charges *not* preferred, and we have nothing to say; but after our having done all any man could do for peace, what must be said of the parties who, instead of appreciating it, opened the war the next morning by a lot of flimsy "charges" against us,—charging us with malice in writing the exposé? We had anticipated such, and of course the "charge" fell through; but the *intent* was there (talk of malice); and this by the man *Burch*, who did us such great wrong and injustice in order to "put himself on our side;" the man who said we "deserved a munching," and that it took such a kingly beast, a "*lion*," to do it "in such a dignified and kingly manner,"—(talk of toadying) ---not in his own defense, but resentment for our supposed attack on his munching lion, the proprietor of Ghent's Hepatic Pills, (dose 1 to 3), whom, he said himself, he did not know personsonally.

We are now "foot-loose;" and while we shall continue to advocate and insist upon a strict conformity to the code, and that physicians, especially members of our organization, shall make their lives morally, socially and professionally shining models for mulation by the younger brethren; insist that in time—it will take time—the State Medical Association shall come to represent only the highest and best type of professional character, we will endeavor to make no personal application of our remarks, and we sincerely hope occasion may not require it. We will fight principles, and not men, and point the way; but should any member depart from the path of rectitude, or be guilty of any act which, in our judgment, is calculated to bring reproach upon the Association, we would not fear or hesitate to denounce it; but we will not presume to dictate, or even to suggest a remedy; the Association is fully competent, if so disposed, to purify its own premises, and, it must be presumed, understands its business.

Do not understand us that we have anything to take back, or any errors to confess. If, however, any injustice has been done any one, we disclaim the intention, and it must be attributed to zeal in the cause. We have not aimed to be personal or "malicious." What we have done or said we believed to be for the best interests—at least for the honor and dignity of the profession, and we shall always believe we were right, and there will be no let up on *quackery*, wherever it may be found. Should any unworthy person get into the Association, however, it shall be made no business of the JOURNAL. It will seek none the less to advance rational medicine in Texas, and so work for the Association that in time all may recognize it as the true exponent of the divine science. We will still work for organization, believing, as we have often said, that until the profession is thoroughly organized all over the State, and working harmoniously together, we will never be able to secure recognition or aid from the State in restricting the right to practice medicine to the hands of those who have been educated and trained to it.

And now, having sacrificed our own interest to that of the cause, [?] and in so doing sacrificed the right of the majority to exexcise a preference, we hold out the olive branch of peace, and say to those who have, through prejudice, engendered, perhaps, by misrepresentation by those unfriendly to us, or through misunderstanding of our true motives and policy—those who have not fairly estimated us, and holding aloof, have not given us their co-operation—join us now; yield your allegiance, and help the JOURNAL in the grand work it has set its hands to do; give us your moral and pecuniary support* in the continued effort to make the medical profession of Texas known and appreciated abroad as the peer of any in the world..

With our kindest thanks to those who have honored us and have been steadfast through all these times of bitter persecution and misrepresentation and wrong, and with our best wishes to the body collectively for its continued prosperity and advancement, we put down the pen of office, and modestly retire to a back seat in the Association.†

*It is most gratifying that many have even anticipated our invitation, and subscribed, the new Secretary setting the example.

†Henceforth none of our personal affairs will appear in these pages.

